# Octominin Inhibits LPS-Induced Chemokine and Pro-inflammatory Cytokine Secretion from RAW 264.7 Macrophages via Blocking TLRs/NF-κB Signal Transduction

**DOI:** 10.3390/biom10040511

**Published:** 2020-03-27

**Authors:** K. K. Asanka Sanjeewa, D. P. Nagahawatta, Hye-Won Yang, Jae Young Oh, Thilina U. Jayawardena, You-Jin Jeon, Mahanama De Zoysa, Ilson Whang, Bomi Ryu

**Affiliations:** 1 Department of Marine Life Science, Jeju National University, Jeju 63243, Korea; asanka@jejunu.ac.kr (K.K.A.S.); dineth1673@gmail.com (D.P.N.); koty221@naver.com (H.-W.Y.); ojy0724@naver.com (J.Y.O.); tuduwaka@gmail.com (T.U.J.); youjinj@jejunu.ac.kr (Y.-J.J.); 2 Marine Science Institute, Jeju National University, Jeju Self-Governing Province 63333, Korea; 3 College of Veterinary Medicine, Chungnam National University, Yuseong-gu, Daejeon 34134, Korea; mahanama@cnu.ac.kr; 4 Department of Genetic Resources Research, National Marine Biodiversity Institute of Korea (MABIK), Chungchungnam-do 33662, Korea

**Keywords:** *Octopus minor*, peptide, inflammation, chemokines, RAW 264.7, NF-κB

## Abstract

Inflammation is a well-organized innate immune response that plays an important role during the pathogen attacks and mechanical injuries. The Toll-like receptors (TLR)/nuclear factor kappa-light-chain-enhancer of activated B cells (NF-κB) is a major signal transduction pathway observed in RAW 264.7 macrophages during the inflammatory responses. Here, we investigated the anti-inflammatory effects of Octominin; a bio-active peptide developed from *Octopus minor* in RAW 264.7 macrophages in vitro. Octominin was found to inhibit lipopolysaccharides (LPS)-stimulated transcriptional activation of NF-κB in RAW 264.7 cells and dose-dependently decreased the mRNA expression levels of TLR4. Specifically, in silico docking results demonstrated that Octominin has a potential to inhibit TLR4 mediated inflammatory responses via blocking formation of TLR4/MD-2/LPS complex. We also demonstrated that Octominin could significantly inhibit LPS-induced secretion of pro-inflammatory cytokine (interleukin-β; IL-1β, IL-6, and tumor necrosis factor-α) and chemokines (CCL3, CCL4, CCL5, and CXCL10) from RAW 264.7 cells. Additionally, Octominin repressed the LPS-induced pro-inflammatory mediators including nitric oxide (NO), prostaglandin E2, inducible NO synthase, and cyclooxygenase 2 in macrophages. These results suggest that Octominin is a potential inhibitor of TLRs/NF-κB signal transduction pathway and is a potential candidate for the treatment of inflammatory diseases.

## 1. Introduction

*Octopus minor* (Sasaki, 1920) is a member of class Cephalopods and phylum Mollusca; known as the common long-arm octopus. Compared to the other octopus species, *O. minor* has a small body, short life spam, and thin arms [1]. *O. minor* is widely distributed along the coastal waters of East Asian countries such as Korea, Japan, and China. *O. minor* is an economically important sea food species and considering as a high nutrition and low-calorie food [2]. In addition to its nutritional value, the fresh and unique flavor of *O. minor* also contribute to the increased demand for it in the seafood market of East Asia. Due to the economic and ecological importance of *O. minor,* it has drawn extensive attention of researchers and *O. minor* is one of the most studied octopods in East Asia [3]. Peptides isolated from different octopus species have also been extensively studied due to their remarkable biological activities against pathological conditions including inflammatory, autoimmune, hormone related, and cardiovascular diseases [4]. However, most of the studies conducted on *O. minor* have been limited to genome studies, co-culture techniques, and nutritional analysis; limited attention have been given to identify its bioactive properties such as anti-inflammatory, anticancer, and antioxidants. It is a well-known fact that the marine ecosystems represent a rich source of structurally unique and novel bioactive compounds from the perspective of potential therapeutic agents [5,6]. Among the marine natural products, peptides are important bioactive natural products which are abundantly found in many marine organisms [7]. Recently, considerable attention has been focused to identify bioactive marine peptides because of their novel chemistry and diverse biological properties Peptides isolated from different organisms have been found to possess interesting bioactive properties such as Reactive oxygen species (ROS) scavenging, preventing lipid peroxidation, antimicrobial, anticancer, antiviral, antihypertensive, antidiabetic, anti-inflammatory, and anticoagulant [7,8]. Taken together, in the present study authors attempted to evaluate anti-inflammatory properties of a novel peptide identified from *O. minor.* Therefore, we designed and synthesized a peptide of 23 amino acids (1-GWLIRGAIHAGKAIHGLIHRRRH-23) from a defense protein 3 cDNA sequence of *O. minor*. The sequence of the peptide, which was designated as Octominin, and was able to act as antimicrobial peptide too.

Inflammation is an evolutionarily conserved complex biological process that occurs in response to interruption of the tissue homeostasis caused by the presence of a biological, physical, or chemical stimulus [9,10]. Under the aforementioned stress conditions, innate and adaptive immune systems co-ordinate to initiate controlled inflammatory responses to control tissue damages or pathogen attacks [11]. However, excessive, uncontrolled, or prolonged inflammatory responses are responsible for the pathogenesis of inflammatory diseases including cancers and tissue damage [12]. During the last decade, the importance of Toll-like receptors (TLRs) during the inflammatory responses were well documented. Specifically, several studies have identified overexpression of TLRs responsible for pathogenesis of chronic inflammatory diseases [13]. According to the literature, there are 13 known TLRs found in mammalian cells. Among these 13 receptors, TLR2 and TLR4 are expressed by macrophages (in response to LPS) found to be associated with development of different inflammatory diseases via activating NF-κB pathway [14].

Specifically, TLR4 is activated by LPS [15]. In detail, LPS is recognized by TLR4 via forming a complex on the cell surface with other specific proteins that are required for ligand recognition [16,17,18,19]. LPS binding protein (LBP) is bound with LPS at the beginning and it causes the moving of LPS to CD14. Moreover, myeloid differentiation factor 2 (MD-2) protein is required for binding of LPS. It is associated with the extracellular domain of TLR4 [20]. LPS is considered to be a glycolipid located in the outer membrane of Gram-negative bacteria. The conserved molecular pattern of LPS is lipid A and it is considered as the main inducer of immunological responses to LPS [21]. As mentioned above, this stimulation process requires two necessary proteins LBP and CD14. The TLR4-MD-2 complex presents as a heterodimer and is expressed ligand specificity. The structurally diverse LPS molecules activate the TLR4 signaling cascade and minor changes of synthetic derivatives of LPS eliminate their endotoxic potency [22,23,24]. The receptor consists of two copies of the TLR4-MD-2. The overall folding of the monomeric structure of the TLR4 and MD-2 complex are not disturbed by LPS binding or dimerization. Moreover, MD-2 contains a β-cup fold structure that consists of two antiparallel β sheets. This structure makes a large hydrophobic pocket and LPS binds to this pocket and causes the above-mentioned dimerization [25,26]. Thus, compounds able to bind with this pocket can causes the steric hindrance to LPS and further activation of inflammatory cascades such as NF-κB, MAPKs, pro-inflammatory cytokines, and chemokines.

The activation of NF-κB transcription factors then triggers the gene transcription related to the inflammatory mediators such as nitric oxide synthase (iNOS), cyclooxygenase (COX2), cytokines (IL-1β, IL-6, and TNF-α), and chemokines (CCL3, CCL4, CCL5, and CXCL10) [27]. It has been reported that pro-inflammatory cytokines such as TNF-α, IL-1α, IL-1β, and IL-6 produced during the inflammatory responses contribute to the pathogenesis of diseases such as degeneration of the intervertebral discs, epilepsy, osteoarthritis, initiation and progression of cancer, depression, and upregulate chemokine secretion from macrophages [28,29,30,31]. Chemokines are small (7 to 13 kDa) heparin-binding proteins, which are found to play an important role during acute and chronic inflammatory responses [32,33]. Furthermore, chemokines such as CCL3 (macrophage inflammatory protein 1α, or MIP-1α), CCL4 (MIP-1β), CCL5 (RANTES), and CXCL10 produced during inflammatory responses primarily act as attractants for leukocytes (monocytes and neutrophils), and are regarded as mediators of chronic and acute inflammation [33]. Recently, a number of studies reported that overexpression of chemokines is responsible for the pathogenesis of disease conditions such as osteoarthritis, liver diseases, and cancers [34,35]. Additionally, the overexpression of iNOS and COX2 in tissues leads to production of NO and PGE2, where those mediators have been demonstrated to play pivotal roles in the development of a number of inflammatory diseases [36].

Previously, several studies have reported that anti-microbial peptides have a potential to act as anti-inflammatory agents via blocking the TLRs mediated NF-κB pathway [37]. Specifically, anti-microbial peptides isolated from *Pichia pastoris* and *Sipunculus nudus* found to inhibit LPS-activated inflammatory responses in macrophages [38,39]. These results suggest that anti-microbial peptides can serve as effective anti-inflammatory agent to treat inflammatory diseases. Thus, in the present study, we attempted to evaluate anti-inflammatory effects of Octominin using LPS-activated RAW 264.7 macrophages.

## 2. Materials and Methods

### 2.1. Chemicals and Regents

Fetal bovine serum (FBS) was purchased from Gibco/BRL (Burlington, ON, Canada). Pen Strep was purchased from life technologies corporation, Grand Island, NY, USA. Enhanced chemiluminescence (ECL) regent was purchased from Amersham, Arlington Heights, IL, USA. The enzyme linked immunosorbent assay kit (ELISA) for prostaglandin E2 (PGE2) (cat# KGE004B) and IL-1β (cat# MLB00C) were purchased from R&D System Inc. (Minneapolis, MN, USA). ELISA kits for mouse TNF-α (cat# 560478), and IL-6 (cat# 550950) were purchased from Becton, Dickinson and Company (BD Biosciences; San Jose, CA, USA). Cytosolic and nucleus proteins extracted from commercial protein extraction kit (#78833) were purchased from Thermo Scientific; Rockford, USA. The following primary antibodies for Western blots: iNOS (#13120), COX2 (#12282), phospo NF-κB-P50 (#4806), NF-κB-P50 (#3035), phospo NF-κB-P65 (#3033), NF-κB-P65 (#8242), nucleolin (#14574), β-actin (#4970), and rabbit secondary antibody (#7074) were purchased from Cell Signaling Technology MA, USA). All analytical grade organic solvents were purchased from Sigma-Aldrich or otherwise mentioned in the text.

### 2.2. Preparation of Samples

Two healthy *Octopus minor* were obtained from Shinan mudflat on the southwest coast of Korea with a mean body weight of ~120 g. Experimental octopus were acclimatized over a week in a 300 L flow-through system tank with the conditions of salinity 34 ± 1 psu and pH 8.0 ± 0.4 at 20 ± 1 °C prior to the experiments. Animals were anesthetized by immersion in 2% ethanol-containing artificial seawater for 5~10 min. When they became relaxed and silent, 13 different tissues (brain, branchial heart, buccal mass, eye, heart, kidney, liver, ovary, poison gland, siphon, skin, and suckers) were excised. Then, all the collected tissues were snap frozen in liquid nitrogen and stored at −80 °C until isolation of RNA. Total RNA was extracted using the RNeasy Mini Kit (Qiagen, Hilden, Germany) according to the manufacturer’s instructions. RNA quality was confirmed using an Agilent 2100 Bioanalyzer (Agilent Technologies, Waldbronn, Germany). All animal procedures were performed in accordance with the Guidelines for Korea Institute of Human Resources Development in Science & Technology (approval ID: 2019-11-30-S-E-697599).

### 2.3. Transcriptome sequencing and synthesis of Octominin

Total RNA was extracted from 13 tissues (eye, brain, branchial heart, liver, buccal mass, heart, kidney, ovary, siphon, poison gland, skin, and suckers) parts of *O. minor* using the RNeasy Mini Kit (Qiagen, Hilden, Germany) following the vendor’s instructions. Purity of extracted RNA was confirmed using an Agilent Bioanalyzer. Isoform sequencing was performed using pooled RNA collected from 13 organs. Library construction and sequencing were performed using PacBio RS II (DNA Link, Inc., Seoul, Korea). On the basis of the N-terminal amino acid sequence of defense protein 3, Octomine (^1^GWLIRGAIHAGKAIHGLIHRRRH^23^) was synthesized as described in a previous report [40].

### 2.4. Molecular Docking

The crystaliographic structure of TLR4/myeloid differentiation factor 2 (MD-2) (PDB: 3FXI) was obtained from the Protein Data Bank (PDB) (http://www.rcsb.org/pdb). The two-dimensional structures of Octominin was drawn by MDL ISIS Draw 2.5 standalone software and converted into three-dimensional structures using the Accelrys Discovery Studio 3.0 (Accelrys, Inc). Binding was evaluated based on CDOCKER.

### 2.5. Cell Culture

RAW 264.7 cells (American Type Culture Collection; ATCC, Manassas, VA, USA) were cultured in Dulbecco’s modified Eagle’s medium (DMEM) (Gibco/BRL; Burlington, ON, Canada) supplemented with 10% heat inactivated FBS and 1% antibiotics in 37 °C at 100% humidity in 5% CO_2_, as previously described [41].

### 2.6. Cell Viability and Dose Range Determination

For the experiments, RAW 264.7 cells were cultured in 24-cell culture plates (45,000 cells/well) and treated with LPS (1μg/mL), alone or in combination with Octominin (62.5, 125, and 250 µg/mL) in a total volume of 500 µL. After 24 h, culture media containing 3-(4, 5-dimethylthiazol-2-yl)-2, 5-diphenyltetrazolium bromide (MTT)(0.1 mg/mL) was added to each well. After 1 h, culture media was removed, and formazan crystals were dissolved in dimethyl sulfoxide (DMSO) and measured at 550 nm using a microplate reader (BioTek Instruments, Vernuski, WI, USA).

### 2.7. Determination of Nitric Oxide, PGE2, and Pro-Inflammatory Cytokine Production

Macrophages were incubated with different concentration of Octominin and LPS for 24 h. The concentration of NO in the culture media was determined by Griess reagent as described by Abekura et al. (2019). The level of PGE2, TNF-α, IL-1β, and IL-6 in culture medium were measured using ELISA assays according to the vender’s instructions [42].

### 2.8. Western Blotting Analysis

Cytosolic and nucleus proteins were extracted with a nuclear and cytoplasmic protein extraction kit using the manufacturer’s instructions (Thermo Scientific; Rockford, USA). An equal amount of protein was electrophoresed in 10% SDS-PAGE. Then, the separated proteins were transferred onto PVDF membranes. Then, PVDF membranes were incubated with primary antibody (1:1000 dilution) at cold room for 8 h. The blots were washed two times with tween 20/Tris-buffered saline and incubated with HRP-conjugated rabbit IgG for 45 min (1:3000) in room temperature. The protein bands on PVDF membranes were detected by using an enhanced chemiluminescent substrate. Membranes were captured using a FUSION SOLO Vilber Lourmat system. Signal intensities of protein bands were determined by densitometry using ImageJ (version 1.4, MD, USA).

### 2.9. RNA Isolation and Reverse Transcription PCR Analysis

RAW 264.7 cells (1.5 × 10^5^ cells/well) were plated into 6-well plates and pretreated with different concentrations (62.5, 125, and 250 µg/mL) of Octominin, then stimulated with 1 µg/mL LPS for 24 h. RAW 264.7 cells were collected and total RNA was isolated using the Trizol regent on the basis of the manufacturer’s instructions. The quantity of total RNA was determined at 260 and 280 nm. Total RNA (5 μg) was reverse transcribed to synthesize cDNA using a first-strand cDNA synthesis kit according to the vender’s instructions (TaKaRa, Tokyo, Japan). Target genes were then amplified by RT-PCR with specific oligo dT primers purchased from (Bioneer, Seoul, Korea). GAPDH used as an internal control. Reactions were carried out in a 10 μL volume containing 3 μL diluted cDNA template, 5 μL of 2 × TaKaRa ExTaq™ SYBR premix, 0.4 μL each of the forward and reverse primers (10 μM), and 1.2 μL ddH_2_O. Briefly, the reaction was performed using the following profile: one cycle at 95 °C for 10 s, followed by 45 cycles at 95 °C for 5 s, 57.5 °C for 10 s, and 72 °C for 20 s, and a final single cycle at 95 °C for 15 s, 55 °C for 30 s, and 95 °C for 15 s. The relative gene expression levels were analyzed by the 2^−ΔΔCT^ method. The data represent as the mean ± standard error (SE) of the relative mRNA expression level from three consecutive studies. The Mann–Whitney U test was used to determine the significance of gene expression levels. The primers used in this study were mentioned in the Table S1.

### 2.10. Statistical Analysis

Results are expressed as means ± S.D. of three independent experiments. Data were analyzed for statistical significance by IBM^®^ SPSS^®^ Statistics version 20 for Windows. Statistical significance was determined by ANOVA followed by Duncan method for multiple comparisons. A value of *p* < 0.05 and 0.01 was considered significant.

## 3. Results

### 3.1. Octominin Repress TLR4/MD-2 Complex Formation In Silico

**Figure 1:**
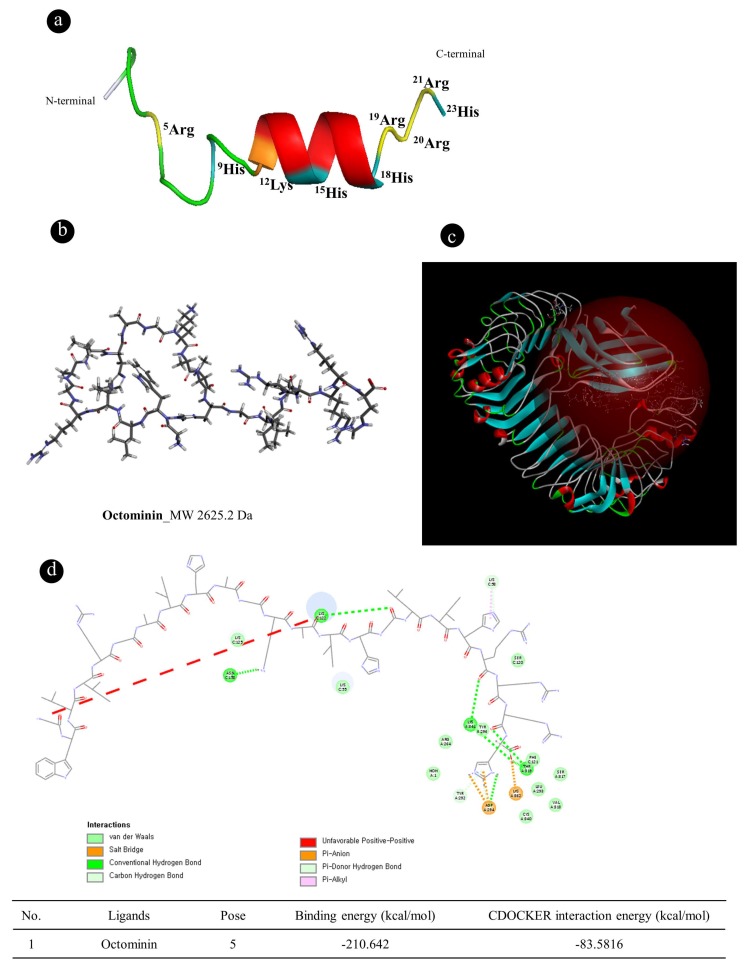
Molecular docking of Octominin to the Toll-like receptor 4 (TLR4)/myeloid differentiation factor 2 (MD-2) complex. (**a**) Three-dimensional (3D) structures of the amino acid sequence of Octominin. Adopted from Nikapitiya et al. (2020); (**b**) Two-dimensional (2D) diagram of Octominin (The most hydrophobic residue is green, and the amount of green is decreasing proportionally to the hydrophobicity, with zero hydrophobicity coded as yellow); (**c**) Predicted Octominin binding site to the crystal structure of the TLR4/MD-2 complex; and (**d**) amino acid binding cites of Octominin with TLR4/MD-complex.

**Figure 2:**
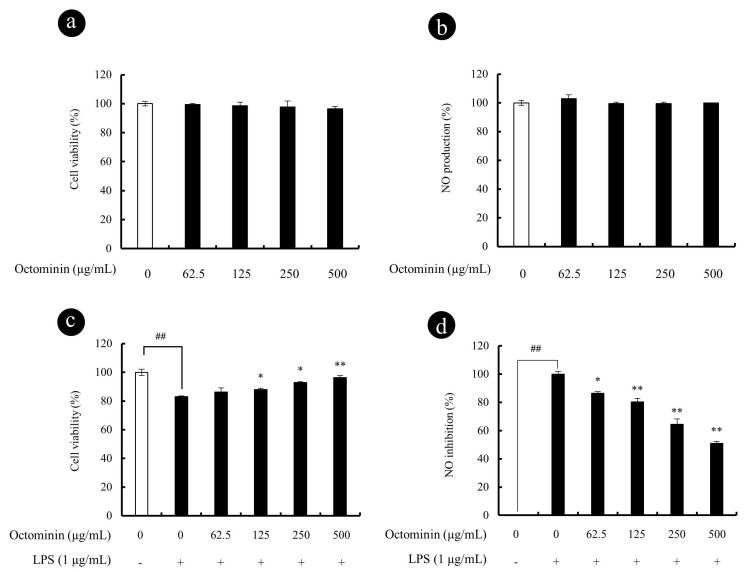
Cyto-protective and NO inhibitory effect of Octominin in LPS-activated RAW 264.7 macrophages. Cytotoxicity (**a**) and NO production (**b**) of Octominin in RAW 264.7 macrophages; Cytoprotective (**c**) and NO inhibitory (**d**) effect of Octominin in LPS-stimulated RAW 264.7 macrophages. Macrophages were seeded in 24-well plates and incubate for 24 h. Then, Octominin (62.5~500 μg/mL) treated to each well and incubate for 1 h and exposed to LPS (1 μg/mL). After 24 h, cells and culture supernatants were used to determine viability (MTT) and NO production, respectively. Experiments were triplicated to evaluate the data, and the mean value is expressed as ±SD. Statistical significance was determined versus vehicle-treated control group (^#^ <0.05 and ^##^ <0.01) or LPS-treated group (* *p* < 0.05 and *p* ** <0.01).

The peptide (Figure 1a) was synthesized according to the previously reported method [28]. Nikapitiya et al. (2020) reported the antifungal action of Octominin. To further confirm the anti-inflammatory effects of Octominin (Figure 1b), observations of LPS-activated macrophages were computationally studied designed to predict its TLR4/MD-2 binding ability. To explore the possible binding modes, Octominin was virtually docked in a 3D model of the TLR4/MD-2 complex using the Accelrys Discovery Studio 3.0 (Figure 1c). As shown in Figure 2b, molecular docking showed that Octominin was fit/move to the pocket of the TLR4/MD-2 complex and interacted with several amino acids sites such as SER120, SER 917, LEU293, LYS941, and THR919 (refer to Figure 1d) in TLR4 which occupied the space and weakened the binding of LPS with TLR4/MD-2.

### 3.2. Octominin Represses NO Secretion in LPS-Activated RAW 264.7 Macrophages without Showing Cytotoxic Effects

Octominin-induced cytotoxicity and NO production is presented in Figure 2. The cell viability of the untreated control group was designated as 100%, indicating no cytotoxicity (Figure 2a). According to the results, the concentrations of Octominin between 62.5 and 500 µg/mL did not show any significant cell viability reduction or NO production (Figure 2b) as compared with the control group after 24 h incubation with RAW 264.7 macrophages. Thus, to determine the protective effects of Octominin, the values between 62.5 and 500 µg/mL were considered as non-toxic to macrophages. The protective effects of Octominin against the LPS-induced cytotoxicity and NO production are presented in Figure 2c,d, respectively. Octominin showed enhanced cyto-protective effect and inhibited NO production in a dose-dependent manner from 62.5 to 500 µg/mL of Octominin in LPS-activated macrophages. Considering amounts required for biological assays, cyto-protective, and NO inhibition data, the authors decided to use 62.5, 125, and 250 μg/mL concentrations of Octominin in consequent studies.

### 3.3. Octominin Repress LPS-Induced PGE2 and Pro-Inflammatory Cytokine Secretion from RAW 264.7 Macrophages

**Figure 3:**
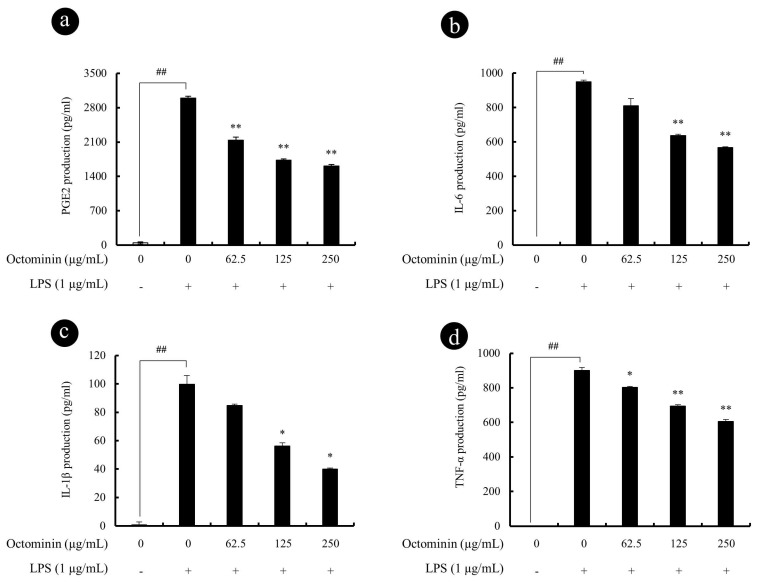
Octominin repress LPS-induced PGE2 and pro-inflammatory cytokine secretion from RAW 264.7 macrophages. Macrophages were exposed to Octominin for 1 h prior to activation with LPS. Cells were incubated for 24 h, and then the cell-free culture supernatants were collected. PGE2 (**a**), IL-6 (**b**), IL-1β (**c**), and TNF-α (**d**) levels in the culture mediums were determined using ELISA kits. Experiments were triplicated to evaluate the data, and the mean value is expressed as ±SD. Statistical significance was determined versus vehicle-treated control group (^#^ <0.05 and ^##^ <0.01) or LPS-treated group (* <0.05 and ** <0.01).

As the next part of study, inhibitory effect of Octominin against LPS-induced pro-inflammatory cytokine and PGE2 secretion in RAW 264.7 macrophages were compared using ELISA assay. As shown in Figure 3, LPS stimulation significantly increased the levels of PGE2, IL-1β, IL-6, and TNF-α in culture supernatants after 24 h. However, treatment with Octominin (62.5~250 μg/mL) prior to LPS activation significantly downregulated the secretion of PGE2 (Figure 3a) and pro-inflammatory cytokines (IL-6 Figure 3b, IL-1β Figure 3c, and TNF-α Figure 3d) from stimulated macrophages as compared wjith the LPS-activated group.

### 3.4. Octominin Represses LPS-Induced Gene Expression of Chemokines and Pro-Inflammatory Cytokine in Activated RAW 264.7 Macrophages

**Figure 4:**
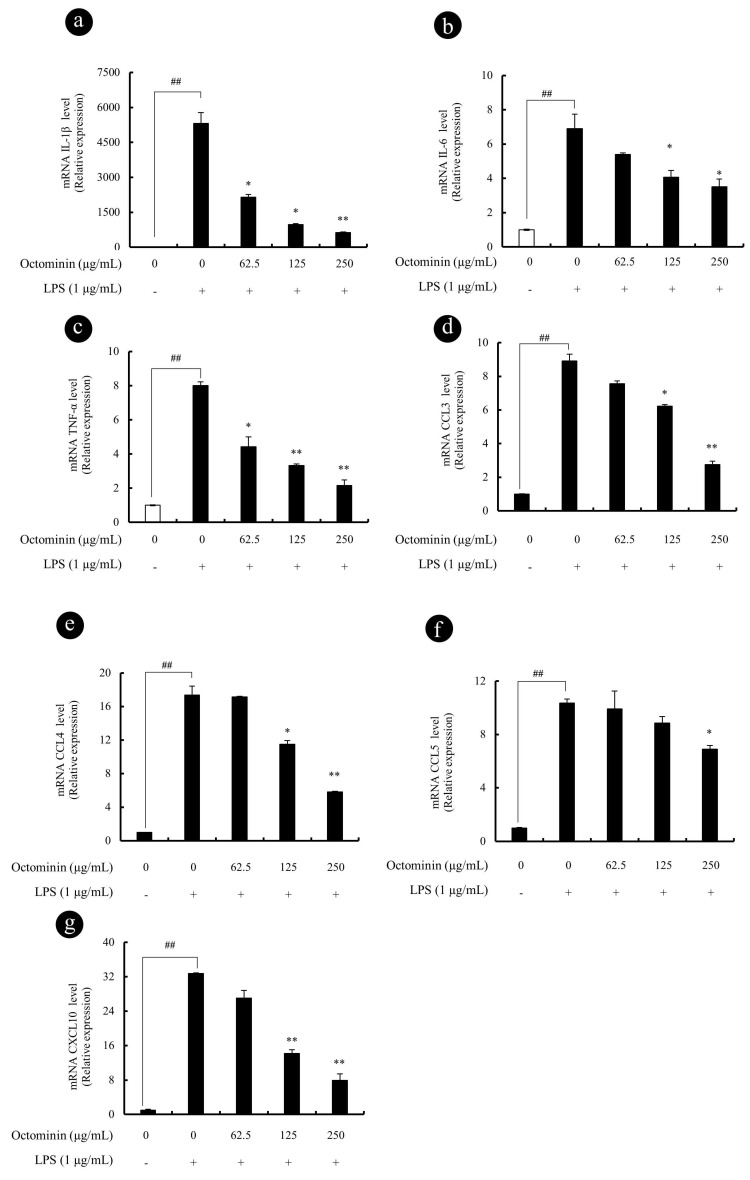
Octominin inhibits the production of pro-inflammatory cytokines and chemokines in the LPS activated RAW 264.7 macrophages. Cells were incubated for 6 h before RNA isolation. Relative expression of IL-1β (**a**), IL-6 (**b**), TNF-α (**c**), CCL3 (**d**), CCL4 (**e**), CCL5 (**f**), and CXCL10 (**g**) was measured using qPCR with GAPDH as the internal reference gene. Experiments were triplicated to evaluate the data, and the mean value is expressed as ± SD. mRNA significance relative to the vehicle-treated control group was calculated using the Mann–Whitney U test (^#^ <0.05 and ^##^ <0.01) or LPS-treated group (* <0.05 and ** <0.01).

In order to determine the effect of Octominin on the transcription of pro-inflammatory and chemokines signaling genes, qPCR analysis of IL-1β, IL-6, TNF-α, CCL3, CCL4, CCL5, and CXCL10 in LPS-activated macrophages was performed. In line with the previous observations, we observed that LPS remarkably promoted the gene transcription of pro-inflammatory cytokines (Figure 4) including IL-1β (Figure 4a), IL-6 (Figure 4b), and TNF-α (Figure 4c) as compared with the vehicle-treated control group. However, Octominin significantly and dose-dependently repressed the elevated cytokine levels observed in LPS-activated macrophages. Furthermore, as shown in Figure 4d,g, the expression of chemokines including CCL3 (MIP-1α), CCL4 (MIP-1β), CCL-5 (RANTES), and CXCL10 was significantly elevated after LPS stimulation, indicating that Octominin significantly and dose-dependently downregulated LPS-induced expression of these chemokines in RAW 264.7 macrophages.

### 3.5. Octominin Represses LPS-Induced iNOS and COX2 Activity in LPS-Activated RAW 264.7 Macrophages

**Figure 5:**
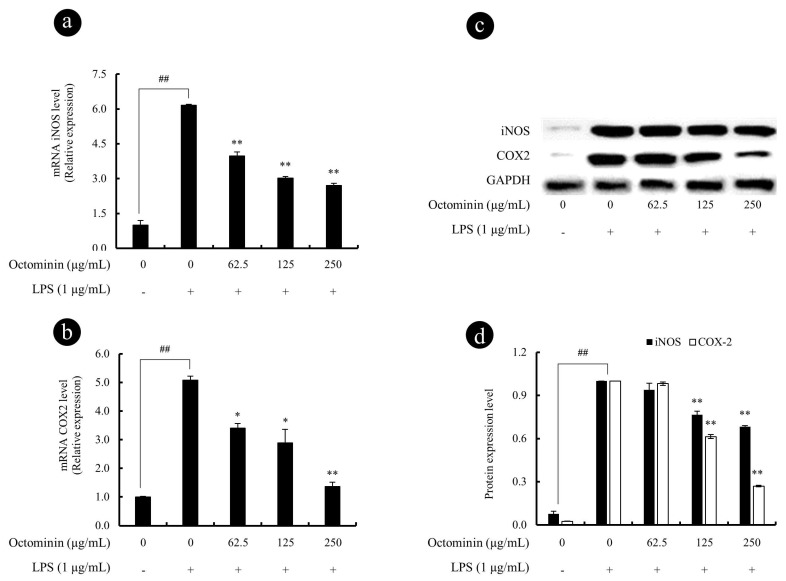
Octominin inhibits the iNOS and COX2 secretion from LPS-activated RAW 264.7 macrophages. The gene expression analysis of iNOS (**a**) and COX2 (**b**) was measured using qPCR with GAPDH as the internal reference gene. The 2^−ΔΔCt^ method was used to calculate the relative mRNA expression levels. Experiments were triplicated. mRNA significance relative to the non-treated control was calculated using the Mann–Whitney U test. Western blots were used to determine the protein expression levels of iNOS and COX2 (**c**), the related expression of the bands was analyzed using ImageJ software (**d**), and β-actin was used as internal control. Results are expressed as the mean ± SD of three separate experiments. Statistical significance was determined versus vehicle-treated control group (^#^ <0.05 and ^##^ <0.01) or LPS-treated group (* <0.05 and ** <0.01).

We next examined the expression of key enzymes responsible for the NO and PGE2 production including iNOS and COX2, respectively. As shown in Figure 5a,b, the mRNA expression levels of iNOS and COX2 were significantly inhibited by Octominin dose-dependently, from activated RAW 264.7 cells. Similar to the mRNA expression results, LPS-stimulated RAW 264.7 cells had upregulated protein levels of iNOS and COX2 (Figure 5c,d), whereas this effect was significantly and dose-dependently inhibited by Octominin (62.5 to 250 μg/mL).

### 3.6. Octominin Represses mRNA Expression Levels of TLRs and NF-κB Phosphorylation in LPS-Activated Macrophages

**Figure 6:**
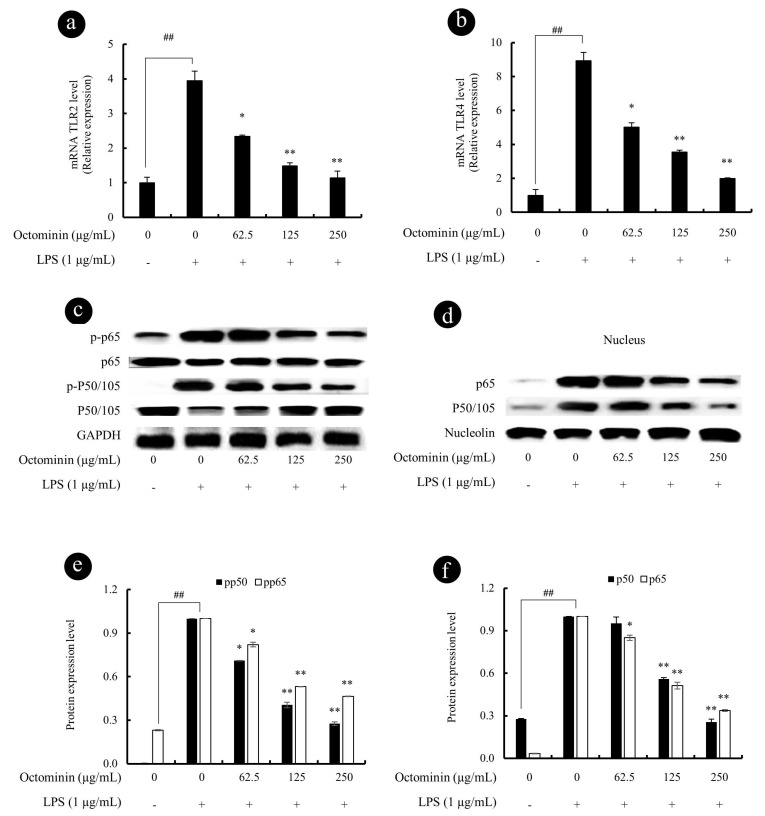
Octominin inhibits the LPS-induced inflammation in macrophages via blocking TLR4/NF-κB signal transduction. The gene expression analysis of TLR2 (**a**) and TLR4 (**b**) was measured using qPCR with GAPDH as the internal reference gene. The 2^−ΔΔCt^ method was used to calculate the relative mRNA expression levels. The translocation of NF-κB p50 and p65 to the nucleus was analyzed by Western blot analysis of cytoplasmic (**c**) and nuclear (**d**) protein extracts. Equal protein loading was controlled using antibodies against β-actin and nucleolin in cytosolic and nuclear extracts, respectively. Relative expression of the bands was analyzed using ImageJ software (**e**,**f**). Results are expressed as the mean ± SD of three separate experiments. Statistical significance was determined versus vehicle-treated control group (^#^ <0.05 and ^##^ <0.01) or LPS-treated group (* <0.05 and ** <0.01).

It is a well-known fact that LPS activates Toll-like receptors (TLRs) and leads to the activation of NF-κB through the recruitment and activation of the upstream proteins [43]. Thus, we examined the mRNA expression levels of TLR2 and TLR4 using qPCR and protein expression levels of NF-κB using Western blots. As shown in Figure 6, the gene expression levels of TLR2 (Figure 6a) and TLR4 (Figure 6b) were significantly upregulated by the LPS treatment as expected. However, Octominin downregulated the elevated TLRs gene expression levels in LPS-activated macrophages. In addition, as shown in Figure 6c,e, the phosphorylation of NF-κB subunit p50 and p65 in the cytosol was significantly increased after the LPS treatment, whereas Octominin significantly downregulated the levels of phosphorylated p50 and p65 in cytosol, suggesting that Octominin has a potential to inhibit NF-κB in cytosol. Nucleus translocation levels of NF-κB p50 and p65 in LPS activated macrophages were also evaluated using Western bolt analysis, as shown in the Figure 6d. According to the results, expression levels of p50 and p65 in LPS-activated macrophages were dose-dependently downregulated by Octominin. However, 62.5 µg/mL of Octominin did not show any significant p50 inhibition in nucleus protein extract (Figure 6f).

## 4. Discussion

**Figure 7:**
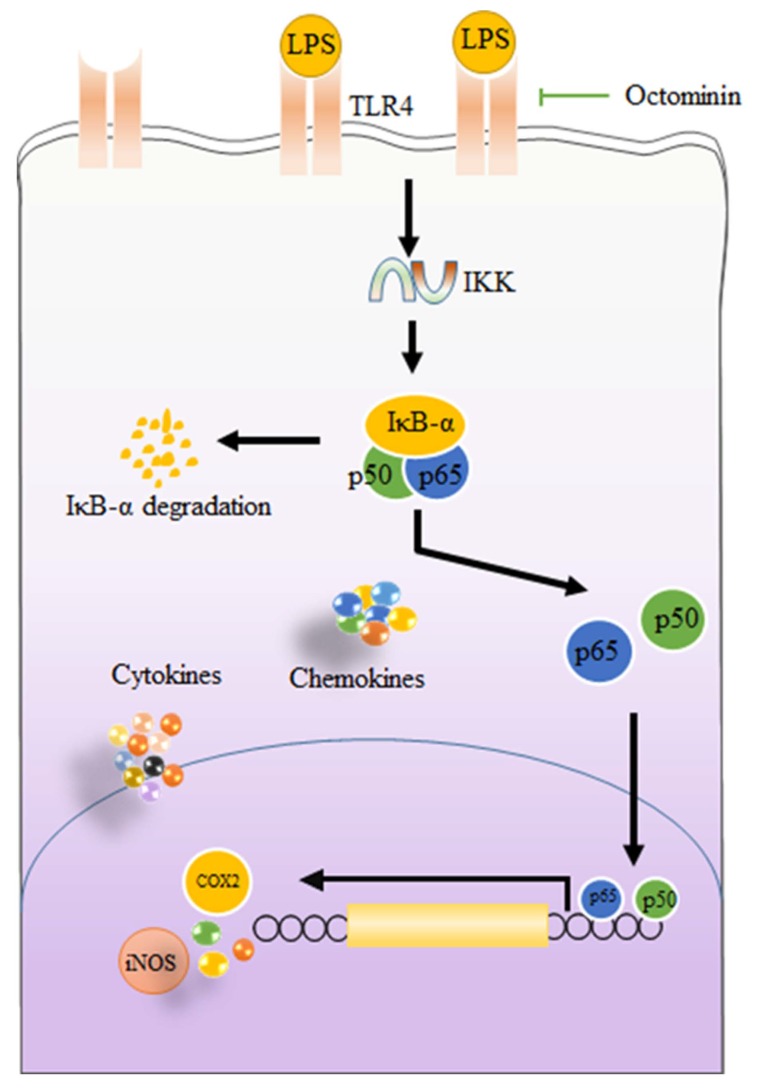
Graphical illustration of possible mechanisms of Octominin to inhibit LPS-activated inflammatory responses in RAW 264.7 macrophages.

Peptides isolated from marine organisms are considered as promising therapeutic agents to treat a range of diseases including antiviral, anticancer, antidiabetic, and antiobesity [4]. Nonetheless, the anti-inflammatory effects and underlying molecular mechanisms of peptides isolated from octopus species are not well understood. In the present study, we report Octominin, a peptide identified from *O. minor*, a common long-arm octopus, can alleviate LPS-activated inflammation via downregulating NO, PGE2, pro-inflammatory cytokines, and chemokines in macrophages. Additionally, we suggest that Octominin can reduce inflammatory responses in LPS-activated macrophages via blocking downsteam activation of TLR/NF-kB signal transduction (Figure 7).

Macrophages play a crucial role during the inflammatory responses. As demonstrated by many studies, LPS-activated macrophages secrete excessive amounts of pro-inflammatory mediators, such as NO and PGE2 [44]. Thus, alternative compounds that inhibit and downregulate the expression of inflammatory mediators can help in treating inflammatory diseases and develop functional products in the future [36]. TLR4 is an important member receptor of TLRs for identify LPS and is responsible to initiate LPS-mediated inflammatory responses. Generally, LPS interacts with hydrophobic pocket in MD-2 and directly links the two components of the TLR4/MD-2/LPS multimer. Lipid chains of LPS (five chains) are submerged in a pocket located in MD-2 and another chain is exposed to the surface of MD-2, forming a hydrophobic interaction with the conserved phenylalanine’s of TLR4 [20]. Therefore, it is essential for LPS to bind with TLR4/MD-2 heterodimer to trigger inflammatory responses [45]. Thus, we evaluated in silico the binding ability of Octominin to the TLR4/MD-2 complex, which could provide insights, as whether Octominin has a potential to act as an anti-inflammatory agent in LPS-activated macrophages. According to in silico docking results, Octominin was buried in the pocket of the TLR4/MD-2 complex by interacting with amino acid sites located in the TLR4/MD-2 heterodimer. This steric hindrance could weaken or reduce the binding of LPS with TLR4/MD-2. Therefore, a series of in vitro experiments were carried out to confirm the anti-inflammatory properties of Octominin.

According to the results of in vitro experiments, Octominin effectively inhibited NO and PGE2 secretion from activated macrophages via downregulating iNOS and COX2 mRNA/protein expression, without reducing cell viability. In addition, Octominin significantly repressed the LPS-induced TNF-α, IL-1β, and IL-6 mRNA expression and protein expression in a dose-dependent manner. Previously, several studies reported similar results as observed in the present study. Specifically, synthesized peptides and antimicrobial peptides (AMP) have been found to exhibit similar anti-inflammatory properties in LPS-activated macrophages. For instance, synthesized peptides and AMP were found to inhibit LPS-induced inflammation in RAW 264.7 macrophages through inhibition of iNOS, COX2, and pro-inflammatory cytokines secretion [46,47]. These results suggest that Octominin exerts anti-inflammatory effects in LPS-activated macrophages by repressing pro-inflammatory mediators.

In the present report, we also noted that Octominin was able to repress LPS-induced chemokines expression from LPS-stimulated RAW 264.7 macrophages including CCL3, CCL4, CCL5, and CXCL10. Chemokines are a group of small proteins secreted by macrophages in response to pro-inflammatory cytokines and they play an important role during the inflammatory responses [48]. In general, chemokines modulate different activities of leukocyte during inflammatory responses such as leucocyte activation, chemotaxis, and recruitment of activated macrophages cell trafficking, cell proliferation, and direction of neutrophils and T cells toward the cite of inflammation [48,49]. Hence, therapeutic compounds targeting chemokine secretion could contribute to inhibiting this inflammatory process. According to the results, the chemokine secretions from activated macrophages were decreased similar to the pro-inflammatory cytokine production from activated macrophages. These results implicate that Octominin is capable of inhibiting LPS-activated inflammatory responses, as well as the development of inflammatory symptoms by reducing leucocyte and neutrophil recruitment to infected areas.

Several studies have demonstrated exposure of macrophages to LPS causes prompt cellular responses. After the reorganization of LPS by pattern receptors such as TLRs, MyD88 is activated. The activated MyD88, subsequently, activates the inhibitor of the kappa B kinase (IKK) complex which is capable of inducing phosphorylation of inhibitor of kappa B (IκB). The phosphorylation of IκB liberates cytoplasmic NF-κB dimers (p50 and p65) and allows them to translocate into the nucleus [16]. Subsequently, translocation of NF-κB facilitates the transcription of genes associated with inflammatory responses in macrophages [50]. Previously, several studies reported that peptides separated from different organisms are capable of inhibiting inflammatory cytokine and chemokines secretion via alleviating NF-κB binding to the nucleus [51]. Here, phosphorylation of p50 and p65 and their nucleus translocations were significantly inhibited by pretreatment with Octominin. As mentioned above, the activation of NF-κB is triggered by LPS after binding to TLRs. The TLR mediated signaling pathways, regulate the release of pro-inflammatory cytokines, as well as chemokines, and are identified as one of the novel therapeutic targets for inflammatory diseases [43]. Therefore, gene expression levels of TLR2 and TLR4 were assessed using RT-qPCR. Similar to the previous studies performed to evaluate anti-inflammatory mechanisms of different peptides, we also noted Octominin has a potential to repress LPS-activated TLRs mRNA expression from LPS-challenged macrophages [52]. Taken together, the results suggested that the anti-inflammatory activity of Octominin is in part mediated through inhibiting NF-κB binding to the nucleus via suppressing TLRs. In summary, a possible mechanism of action by which Octominin modulates LPS-activated inflammatory responses in RAW 264.7 macrophages is shown in Figure 7.

## 5. Conclusions

In conclusion, our results indicated that Octominin could potently alleviate LPS-activated inflammation through TLR/NF-κB axis in RAW 264.7 cells. Furthermore, Octominin suppressed the expressions of iNOS and COX2, as well as pro-inflammatory cytokines (TNF-α, IL-1β, and IL-6), and chemokines (CCL3, CCL4, CCL5, and CXCL10). Subsequent studies demonstrated that Octominin effectively inhibited NF-κB binding via suppressing TLR activation in LPS-activated cells; which could lead to gene transcription of cytokines and chemokines. This study showed that Octominin could be a promising therapeutic agent to be developed as a functional material to treat inflammatory diseases. However, future in vivo studies with Octominin to confirm these initial observations are necessary and opportune.

## Supplementary Materials

The following are available online at https://www.mdpi.com/2218-273X/10/4/511/s1.
