# Supplementary material for: Octominin Inhibits LPS-Induced Chemokine and Pro-inflammatory Cytokine Secretion from RAW 264.7 Macrophages via Blocking TLRs/NF-κB Signal Transduction
Source: Biomolecules. 2020 Mar 27;10(4):511. doi: 10.3390/biom10040511 (PMC7226457; doi:10.3390/biom10040511)
Supplement: Supplementary file 1 [file biomolecules-10-00511-s001.pdf]

Table 1. Primer sequences and efficiency of primers used for the study.

Gene	Primer	Sequence (5′ → 3′)	Efficiency (%)
GAPDH	Sense<br>Antisense	AAGGGTCATCATCTCTGCCC<br>GTGATGGCATGGACTGTGGT	103.47
iNOS	Sense<br>Antisense	ATGTCCGAAGCAAACATCAC<br>TAATGTCCAGGAAGTAGGTG	99.35
COX2	Sense<br>Antisense	CAGCAAATCCTTGCTGTTCC<br>TGGGCAAAGAATGCAAACATC	100.04
IL-1β	Sense<br>Antisense	CAGGATGAGGACATGAGCACC<br>CTCTGCAGACTCAAACTCCAC	96.49
IL-6	Sense<br>Antisense	GTACTCCAGAAGACCAGAGG<br>TGCTGGTGACAACCACGGCC	100.27
TNF-α	Sense<br>Antisense	TTGACCTCAGCGCTGAGTTG<br>CCTGTAGCCCACGTCGTAGC	101.82
TLR2	Sense<br>Antisense	CAGCTGGAGAACTCTGACCC<br>CAAAGAGCCTGAAGTGGGAG	94.39
TLR4	Sense<br>Antisense	CAACATCATCCAGGAAGGC<br>GAAGGCGATACAATTCCACC	94.58
CCL3	Sense<br>Antisense	CGGAAGATTCCACGCCAATTCATCG<br>CAGATCTGCCGGTTTCTCTTAGTCAGG	106.45
CCL4/MIP-1	Sense<br>Antisense	CAGCTCTGTGCAAACCTAACCC<br>AACCCTGGAGCACAGAAGGC	101.37
CCL5/ RANTES	Sense<br>Antisense	TGTTTGTCACTCGAAGGAACCG<br>TGGGGGTCAGAATCAAGAAACCC	109.70
CXCL10	Sense<br>Antisense	ATGACGGGCCAGTGAGAATGAGG<br>GCACTGCACAAGAAGATGCG	104.39
